# Involvement of MicroRNA-210 Demethylation in Steroid-associated Osteonecrosis of the Femoral Head

**DOI:** 10.1038/srep20046

**Published:** 2016-01-25

**Authors:** Heng-feng Yuan, Von Roemeling Christina, Chang-an Guo, Yi-wei Chu, Rong-hua Liu, Zuo-qin Yan

**Affiliations:** 1 Department of Orthopedics, Zhongshan Hospital, Fudan University, Shanghai, China; 2 Department of Cancer Biology, Mayo Clinic, Florida, USA; 3 Mayo Graduate School, Mayo Clinic, Rochester, USA; 4 Department of Immunology, School of Basic Medical Science, Fudan University, Shanghai, China

## Abstract

Angiogenesis is an important event in steroid-associated osteonecrosis of the femoral head (SONFH). Here we performed miRNA microarray with SONFH tissues (ONs) and the adjacent normal tissues (NLs) to select the angiogenic miRNA. The results showed that miR-210 was differentially expressed in SONFH versus normal tissues. Unexpectedly, its specific transcription factor, hypoxia-inducible factor-1α, was shown of no significant changes in ONs compared with NLs. Further Bisulfite sequencing revealed that miR-210 is embedded in a CpG island and miR-210 gene has 2 CpG sites with lower methylation percentage in ONs compared with NLs. Additionally, ONs with lower miR-210 gene methylation exhibited higher miR-210 expression. Next, we found that the endothelial cells treated with demethylating agents could significantly increase the expression of miR-210, along with promoted cell viability and differentiation. Some angiogenic genes (VEGF, bFGF, TNF-α and PCNA) were up-regulated as well. In addition, the supernatant of the cells after demethylation treatment displayed an enhanced ability of recruiting new microvessels *in vivo*. Taken together, our study not only provides novel insights into the regulation of angiogenesis in this disease, but also reveals a therapeutic opportunity for treatment of SONFH patients with demethylating agents.

Corticosteroids have been widely employed to effectively treat autoimmune diseases such as rheumatoid arthritis, systemic lupus erythematosus and nephrotic syndrome^1^,^2^,^3^. Unfortunately, one of the most common and severe complications following corticosteroid treatment is steroid-associated osteonecrosis of the femoral head (SONFH)^4^. The pathogenic mechanisms of SONFH are correlated with impaired vascularization of the femoral head, resulting in partial ischemia and hypoxic status, followed by development of osteonecrosis^4^,^5^. While it is generally believed that the reparative process would be initiated upon discontinuation of treatment via re-activation of angiogenesis, such mechanisms are still poorly understood.

Epigenetics is the study of heritable changes in gene expression without altering the DNA sequence^6^. DNA methylation is one of the most common forms of epigenetic modulation that involved in various chronic diseases^7^,^8^,^9^. It always occurs on a cytosine in a CpG dinucleotide in particular, and is implemented by the addition of a methyl group to the 5 position of a cytosine ring^10^,^11^. In general, the promoter hypermethylation is often associated with gene repression^12^, while the gene body methylation generally leads to increased transcriptional activity^13^ and splicing regulation^14^.

In addition, recent studies suggest that microRNAs (miRNAs) could enter the nucleus^15^ and be associated with establishing DNA methylation^16^,^17^. The miRNAs have been demonstrated to be involved in various physiological and pathological processes^18^. They expressed in a tissue- or cell- specific manner and function through regulating gene expression at the post-transcriptional level^18^,^19^. The epigenetic regulation of miRNAs is a novel area of research in skeletal system diseases^20^,^21^. Previous report has discussed the crosstalk between DNA methylation and the miRNA machinery in the pathogenesis of rheumatoid arthistis^22^. As in the context of SONFH, however, such mechanisms remain to be elucidated.

In this study, we initially screened for specific angiogenic related miRNAs that are associated with SONFH. We then investigated the methylation status of target genes association. After that, we discussed the alteration of angiogenesis after DNA-demethylating agent treatment *in vitro* and *in vivo*.

## Results

### Screening for differentially expressed miRNAs

**Figure 1:**
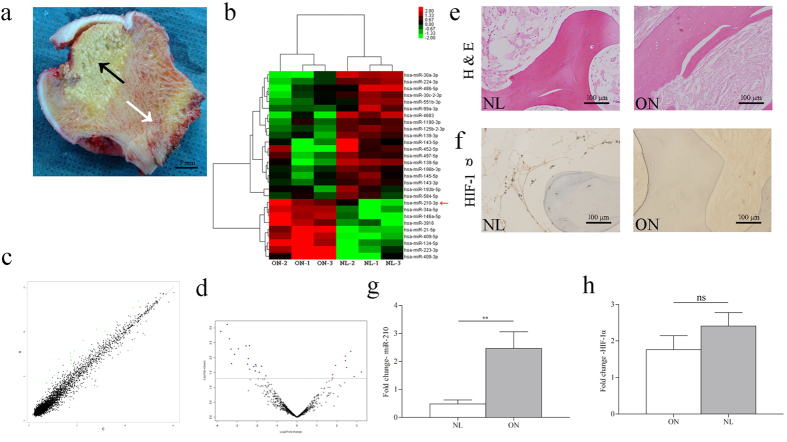
MiR-210 was filtered and validated in patient samples with SONFH. (**a**) Two zones of samples were obtained, which were the osteonecrosis tissues (ONs, black arrow) and the matched normal tissues (NLs, white arrow). (**b–d**) Clustering, heatmap and volcano analysis of the miRNAs differentially expressed in ONs and NLs after the microarray. (**e**) Hematoxylin and eosin staining of the tissues, typical sign of empty lacunae was observed in ONs. (**f**) Immunohistochemistry staining for miR-210 specific regulator, HIF-1α. (**g**) The expression of miR-210 detected by microarray was confirmed by Q-PCR in the extended patient samples. (**h**) The expression of HIF-1α was examined by Q-PCR. *P < 0.05, **P < 0.01.

Three pairs of SONFH tissues (ONs) and adjacent normal tissues (NLs) were screened using a miRNA microarray assay containing 2578 human miRNA sequences. The results demonstrated that 28 miRNAs were differentially expressed between the two groups, of which 9 were up-regulated and 19 were down-regulated in ONs (P < 0.05 with fold-change >3; Supplementary Table S2) (Fig. 1b–d). Among the differentially expressed miRNAs, the up-regulation of miR-210-3p (miR-210) was selected as the target miRNA for further study due to its known function in the up-regulation of angiogenesis in the context of ischemic diseases^23^,^24^.

### Verification of miR-210 and HIF-1α

Hematoxylin and eosin (H & E) staining depicted the ONs with a typical sign of empty lacunae or ghost nuclei in the lacunae surrounded by fibrous tissue (Fig. 1e). Using Quantitative PCR (Q-PCR), miR-210 was found to be significantly up-regulated in the ONs (P < 0.01) when compared with NLs (Fig. 1g) in the extended human samples. Subsequently, we analyzed the expression of HIF-1α (Fig. 1f,h), which is reported to be a crucial regulator of miR-210^24^,^25^, with the methods of Q-PCR and Immunohistochemistry (IHC). However, the results showed that there was no significant difference of HIF-1α expression between ONs and NLs.

### DNA demethylation of miR-210

**Figure 2:**
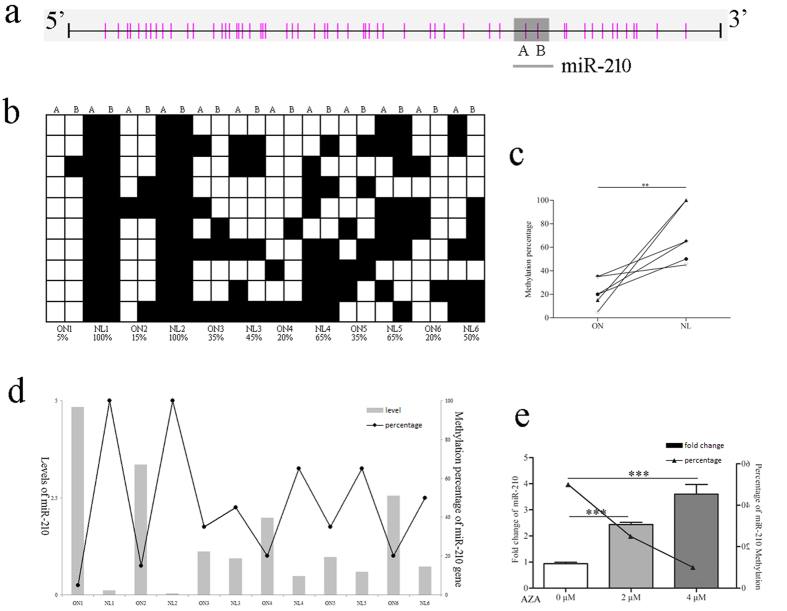
MiR-210 demethylation was involved in SONFH. (**a**) The 539-bp DNA fragment in the promoter region of miR-210 contains 56 CpG sites. Each vertical bar illustrates a single CpG site, and CpG sites A and B are inside the region of miR-210 gene (shown as the grey box). (**b**) Bisulfite sequencing PCR analysis of the 2 miR-210 CpG sites. Ten single clones were represented for each sample (n = 6). Black and white squares represent the methylated and unmethylated CpGs, respectively. (**c**) Methylation percentage of ONs versus NLs. (**d**) The correlation between miR-210 levels (bars) and miR-210 methylation levels (lines) was analyzed in the 6 pairs of human samples. (**e**) HUVECs were exposed under normoxia for 48 h in the presence or absence of AZA, and analyzed the expression (bars) and methylation percentage (lines) of miR-210. **P < 0.01, ***P < 0.001.

We used bisulfite sequencing PCR (BSP) to examine the methylation status of CpG island in the ONs and matched NL tissues (n = 6), which were the same samples used in the miRNA microarray validation. Analysis with Methyl Primer Express v1.0 revealed that miR-210 is embedded in the CpG island of its promoter region, with 2 CpG sites inside its sequence (Fig. 2a). We found that the methylation status of the 2 CpG sites changed significantly, with the percentage of methylation decreased to 21.7% in ONs compared with 70.8% in NLs (P < 0.01) (Fig. 2b,c). Moreover, ONs with lower miR-210 gene methylation exhibited higher miR-210 expression (Fig. 2d).

We further analyzed the expression and methylation changes of miR-210 in human umbilical vein endothelial cells (HUVEC) treated with 5-aza-2′-deoxycytidine (AZA), a DNA methyltransferase inhibitor. As shown in Fig. 2e, the expression of miR-210 was significantly increased with higher doses of AZA (P < 0.001) while the methylation percentage was decreased contrarily.

### *In vitro* study of miR-210 demethylation in angiogenesis

**Figure 3:**
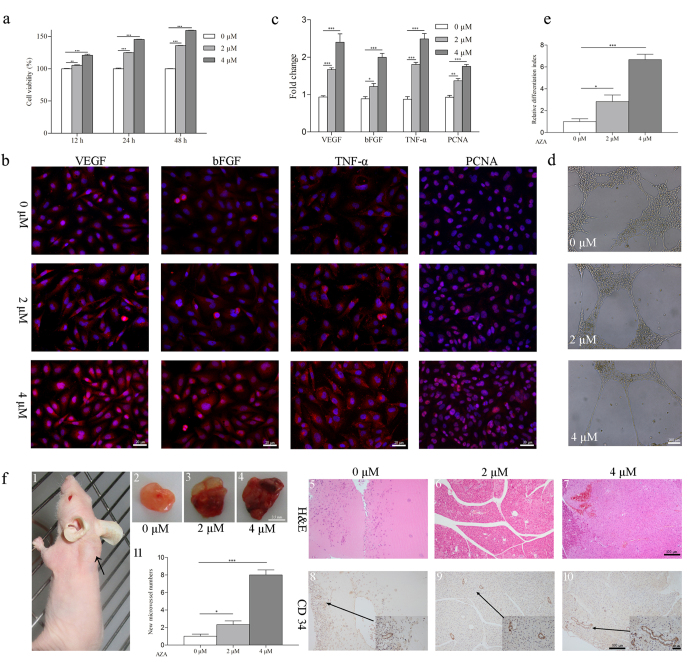
Effect of miR-210 demethylation on angiogenesis. (**a**) HUVECs were treated with AZA (0, 2, or 4μM) for 12, 24, or 48 hours, and proliferation of HUVECs were determined by MTT-based cell proliferation. (**b,c**) Immunofluorescent assay and Q-PCR were performed to examine the levels of angiogenic proteins and genes, which were VEGF, bFGF, TNF-α and PCNA, respectively. (**d,e**)The tubelike structures were identified and quantification of the endothelial cell differentiation was calculated. (**f**) The HUVEC cell culture supernatant mixed with Matrigel was injected into the nude BALB/c mice (**f1**). The plug was removed after two weeks (**f2–4**) and stained with H&E (**f5–7**) and IHC (**f8–10**) to observe the angiogenesis. Meantime, the new microvessel numbers were analyzed (**f11**). *P < 0.05, **P < 0.01, ***P < 0.001.

Considering miR-210 is well known in angiogenesis^24^, we explored the alteration of angiogenic effect after AZA treatment. HUVECs treated with AZA showed a higher viability (Fig. 3a). We further investigated the expression of angiogenic genes in response to AZA. Results showed that after AZA treatment, cells demonstrate significant up-regulation of VEGF, bFGF, TNF-α and PCNA (Fig. 3b,c). Finally, we examined the ability of HUVEC cells to vascular differentiation. We found that HUVECs treated with AZA were more likely to form tube-like structures (Fig. 3d,e), which were the similar characteristics of these cells when stimulated with pro-angiogenic factors such as VEGF and FGF^26^,^27^. Taken together, these results indicate that HUVECs treated with AZA demonstrate enhanced angiogenesis via increased cell viability and differentiation, as well as up-regulation of some pro-angiogenic factors.

### *In vivo* study of miR-210 demethylation in angiogenesis

A Matrigel plug assay was conducted to evaluate the in-growth of new blood vessels *in vivo*. Supernatant of HUVEC cells treated with AZA was collected and incorporated into the matrigel plugs which were then subcutaneously implanted in BALB/C nu/nu mice. Results showed that treatment with AZA markedly enhanced endothelial cell recruitment into the matrigel plug (Fig. 3f). In addition, AZA treated groups demonstrated an increase in the level of CD34 staining (Fig. 3f), a marker for hematopoietic progenitor and small vessel endothelium cells responsible for neovascularization in a variety of tissues^28^. The results suggest that changes of miR-210 in the endothelial cells could recruit the new blood vessels, which is an important mechanism for the repair of SONFH.

## Discussion

The regulation of miRNA expression is critical for regulation of gene expression in normal and pathological processes^29^,^30^. In this study, we performed a miRNA microarray screen with normal and SONFH tissues in an effort to identify expression discrepancies, in which a focus on angiogenic related signaling was employed. Our study identified 28 miRNAs exhibiting more than 3-fold changes in ONs as compared to NLs, of which 19 miRNAs down-regulated and 9 miRNAs up-regulated. Of these, we found that miR-210 was significantly up-regulated in SONFH tissue. These results are consistent with Yamasaki *et al.* study^31^, who reported miR-210 was present in cells surrounding regions of osteonecrosis.

MiR-210 has been previously reported to be up-regulated in a variety of ischemia diseases^23^,^25^,^32^,^33^,^34^. Lou *et al.* found that up-regulation of miR-210 could activate the Notch signaling pathway, which may contribute to angiogenesis after cerebral ischemia^35^. Recently, another study demonstrated that miR-210 could significantly promote the expression of VEGF in bone marrow mesenchymal stem cells in a time-dependent manner^36^. In addition, Usman and his colleagues reported that single local injection of synthetic miR-210 could promote achilles tendon healing in the early phase with the up-regulation of VEGF, FGF2 and type I collagen^37^. These studies indicate that miR-210 may also exert a considerable role in SONFH during the reparative process through the activation of angiogenesis.

The ischemic pathogenesis of SONFH would cause regional hypoxia in the femoral head^38^,^39^, which could be used to explain the up-regulation of miR-210^40^. However, in our study, we observed no significant difference of HIF-1α expression between ONs and NLs. One possible reason could be miRNAs can stably exist for a long time^41^ in ONs while HIF-1α would disappear during the long disease course. Nevertheless, the expression of miRNAs could also be impacted by epigenetic changes in the genome. Recent reports have shown the expression of miR-210 was also regulated by DNA methylation in cardiovascular and gastric diseases^42^,^43^. Our group sought to determine whether similar mechanisms were involved in SONFH, providing novel insight into the regulation of angiogenesis in this disorder.

Our findings demonstrated that miR-210 was hypermethylated in normal bone tissue as compared to SONFH, likely causing suppression of miR-210 expression in these tissues. However, once the osteonecrosis happens to the femoral head, the self-reparative mechanism would be switched on and miR-210 demethylated to up-regulate the expression of miR-210, which could activate the angiogenesis accordingly for the femoral head healing. The epigenetic changes during the reparative process, unlike what happens in tumors, might be the protective consequence of biological evolution.

The HUVECs were selected for further study due to previous report displayed miR-210 expressing cells were mainly endothelial cells in the osteonecrosis tissue^31^. AZA is a DNA methyltransferase inhibitor, and has been approved by the US Food and Drug Administration (FDA) for the treatment of myelodysplastic syndrome^44^,^45^. Here we used AZA to modify miR-210 methylation in HUVECs. The results showed that the expression of miR-210 in HUVECs could be increased owing to decreased miR-210 methylation percentage after AZA treatment, leading to enhanced cell viability and vascular differentiation, as well as the up-regulation of some angiogenic proteins. Our study was consistent with the previous report, which found DNA demethylation could up-regulate the miR-210 expression in neural progenitor cells under normoxia^46^. Moreover, we demonstrated that supernatants of HUVECs treated with AZA could promote endothelial cells and hematopoietic progenitor cells recruit into matrigel plugs that planted inside the mice. Taken together, these results suggest that miR-210 demethylation could regulate the angiogenesis, and participate in the pathophysiological mechanisms of SONFH.

Although it’s unclear how many miRNAs will be regulated in the endothelial cells after AZA treatment, our study showed the increased expression of miR-210. Furthermore, these data suggest that treatment with AZA is possible to facilitate neovascularization in tissues surrounding regions of osteonecrosis, thereby promoting tissue viability and reducing the progression of SONFH. While AZA has been approved by the FDA for the treatment of several cancers and other myelodysplastic syndromes, significant adverse effects have been reported^45^,^47^,^48^. Optimization of dosing and administration may need to be performed to ensure safety and efficacy of this treatment in patients with SONFH. Other DNA methyltransferase inhibitors may also provide some therapeutic benefit similar to AZA.

In conclusion, our results demonstrate that demethylation of miR-210 is involved in the pathogenesis of SONFH. The findings suggest that miR-210 demethylation could serve as a potential therapeutic target for the treatment of SONFH via alteration of angiogenesis.

## Materials and Methods

### Patients

Femoral heads were obtained from 9 patients who had corticosteroid usage histories and fulfilled the diagnosis of ONFH according to the guidelines of the Chinese Medical Association^49^ who were undergoing total hip arthroplasty at Zhongshan Hospital (Shanghai, China). The characteristics of the patients were listed in Supplementary Table S1. Femoral heads were cut along the coronal plane to differentiate the osteonecrosis zone and normal zone (Fig. 1a), which were defined as a pair group. After that, tissues of each zone were cut into small pieces of approximately 5 × 5 × 5 mm^3^, and stored in a −80 °C freezer. This study was carried out in accordance with the approved institutional guidelines, and the protocol was reviewed and approved by Ethical Committee of Zhongshan Hospital (No. B2013-124). Informed consent was obtained from all patients.

### MicroRNA microarray

The procedure for the preparation of samples can be found in our previous report^50^. All samples met the quality control standards. Total RNA from 3 pairs of SONFH patients were hybridized to the Affymetrix GeneChip miRNA 4.0 Array containing 2578 human miRNA sequences. RVM t-test was applied to filter the differentially expressed miRNAs.

### Validation of target miRNA (miR-210) and HIF-1α

The samples from another 6 SONFH patients were used for validation. Q-PCR was applied to detect the expression of miR-210-3p (miR-210) and HIF-1α. Details of the procedure can be found in our previous report^50^. Briefly, RNA-tailing and primer-extension PCR method was used for miR-210 detection using a miRNA assay kit (RiboBio Co., Guangzhou, China) according to the manufacture’s protocol. IHC for HIF-1α protein expression was also performed. The primary antibodies and the secondary antibodies were supplied by Goodbio Technology Co. (Wuhan, China).

### Bisulfite sequencing PCR (BSP)

Genomic DNA from the 6 SONFH patients used for validation was isolated with a Genomic DNA kit (TransGen Biotech, Beijing, China). The DNA was then subjected to bisulfite conversion and purification with the EZ DNA Methylation-GoldTM Kit (Zymo Research Co., CA, USA) according to the manufacturer’s instructions. The BSP primers used were listed in Supplementary Table S1. Amplified PCR products were analyzed in 2% electrophoresis agarose gel and cloned into pMD19-T (TaKaRa Co., Japan). 10 clones were analyzed using colony PCR and positive clones were sequenced (Sangon Biotech Co., Shanghai, China). Percentage of methylation was calculated comprehensively and comparatively by CpG viewer, QUMA and Biq-analyzer.

### *In vitro* study of miR-210 demethylation

HUVECs were a gift provided by Dr. Wang from Fudan University. The cells were treated with a 0, 2 or 4 μM DNA-demethylating agent (AZA) under normoxia for 48h. The genomic DNA was extracted and analyzed the percentage of methylation following the above BSP procedures. The total RNA was extracted, and the level of miR-210 expression was measured with Q-PCR. The cell viability was estimated by MTT assay (MTT, Amresco) at 24h, 48h and 72h. Immunofluorescent assay and Q-PCR were performed to examine the angiogenic proteins and genes including VEGF, bFGF, TNF-α and PCNA. The VEGF and PCNA antibodies were supplied by Boster Co., and bFGF and TNF-α by Abcam. For quantification of endothelial differentiation, we followed Saeid *et al.* method^51^: identified the tubelike structures (≥30mm) and calculated the total tube length from 4 randomly chosen fields. The total area of the culture surface covered by HUVECs was determined in the same fields as well. The differentiation index was the ratio of the total tube length over cell area for each field.

### *In vivo* study of miR-210 demethylation

Fifteen six-week-old BALB/C nu/nu mice were purchased from SLAC Experimental Animal Co., Ltd (Shanghai, China) and maintained under SPF condition. All the animal procedures were approved by the Animal Experimentation Ethics Committee of Fudan University under protocol #DF123 and performed in accordance with institutional policies. The Matrigel (BD, USA) (300 ul) was mixed with the supernatant (100 ul) of the HUVECs from each group, which was collected 24 hours after the cell split. The mixture was then injected subcutaneously into the mice to examine the new microvessel formation. After 2 weeks, the mice were sacrificed and the plugs were removed. Matrigel plugs were stained with H & E staining and analyzed by IHC for mouse CD34 (Abcam).

### Statistical analysis

All data were presented as the mean ± SD if possible, and analyzed using the SPSS16.0 software (Chicago, IL, USA). The student unpaired *t* test or unpaired *t* test with *Welch’s* correction was used to analyze the results between two groups, ANOVA was used to analyze more than two groups. P values < 0.05 were considered to be statistically significant.

## Additional Information

**How to cite this article**: Yuan, H.-F *et al.* Involvement of MicroRNA-210 Demethylation in Steroid-associated Osteonecrosis of the Femoral Head. *Sci. Rep.* **6**, 20046; doi: 10.1038/srep20046 (2016).

## Supplementary Material

Supplementary Table S1

Supplementary Table S2
